# An MPL W515L mutation in refractory anemia with ringed sideroblasts associated with marked thrombocytosis: A case report

**DOI:** 10.3892/ol.2014.2754

**Published:** 2014-12-02

**Authors:** LIN HAO, SANDEEP SEN, DHIVYA SUGUMAR

**Affiliations:** 1 Department of Internal Medicine, Saint Mary’s Health Center, St. Louis, MO 63117, USA; 2 SSM Cancer Care, Saint Mary’s Health Center, St. Louis, MO 63117, USA

**Keywords:** refractory anemia with ringed sideroblasts associated with marked thrombocytosis, myelodysplatic/myeloproliferative neoplasm, MPLW515L, prognosis, mutation

## Abstract

The current study presents the case of a 63-year-old patient exhibiting refractory anemia with ringed sideroblasts associated with marked thrombocytosis (RARS-T), who was positive for the MPL W515L mutation, but negative for the JAK2 V617F mutation. Following diagnosis, the patient remained asymptomatic for over three years, however, in August 2012, the patient relapsed and was administered with supportive treatment in the form of subcutaneous darbepoetin α at a dose of 300 μg/week, which resulted in an increased hemoglobin concentration, allowing the patient to remain transfusion-independent. The MPL W515L mutation has been reported in two previous cases of myelodysplastic/myeloproliferative neoplasms (MDS/MPN) with ringed sideroblasts, however, to the best of our knowledge, the current report is the first to present a case of RARS-T with an MPL W515L mutation. A clinical trial designed to evaluate the efficacy of a targeted agent against the JAK2 V617F mutation is currently ongoing, with the aim of providing a novel therapeutic strategy for treating MDS/MPN patients. As MPL is located upstream of the JAK-STAT signaling pathway, it is a possible therapeutic target in MDS/MPN patients positive for an MPL W515L mutation, but negative for a JAK2 V617F mutation.

## Introduction

The 2008 World Health Organization (WHO) Classification of Tumors defines refractory anemia with ring sideroblasts associated with marked thrombocytosis (RARS-T) as a provisional entity, with unclassifiable myelodysplastic/myeloproliferative neoplasm (MDS/MPN) status, as opposed to a confirmed entity (1). Although thrombocytosis is a poor prognostic factor in MDS patients (2), overall, RARS-T patients exhibit a more favorable prognosis. The JAK2 V617F mutation has been identified in ~50% of RARS-T patients and in only 2/89 cases of typical MDS, indicating that RARS-T should be considered as a JAK2 mutation-associated chronic MPN (3).

RARS-T is characterized by MDS characteristics and <5% blasts in the bone marrow, and is differentiated from other diseases by the presence of ≥15% ringed sideroblasts, thrombocytosis and a platelet count of >450×10^9^/l (1). Clinical manifestations of RARS-T include symptoms associated with anemia, leucopenia and abnormalities of platelet function and quantity, for example, fatigue, infection, bleeding and/or thrombosis. The International Prognostic Scoring System (IPSS) (4), which is commonly used for MDS, is also applicable as a prognostic tool for RARS-T. The management of RARS-T is largely supportive, including transfusion support in patients exhibiting symptomatic anemia and prophylaxis, and treatment of thromboembolism. Similar to MDS, RARS-T patients exhibiting anemia and low serum erythropoietin levels may benefit from the administration of erythropoiesis-stimulating agents. An ongoing clinical trial is currently studying the efficacy of ruxolitinib, an oral JAK2 inhibitor, in JAK2 mutation-positive RARS-T patients (ClinicalTrials.gov Identifier: NCT01895842; http://clinicaltrials.gov/show/NCT01895842). Furthermore, a case study has recently documented the successful treatment of young RARS-T patients with lenalidomide (5).

MPL (6), a cellular homologue of the oncogene v-mpl, belongs to the hematopoietic cytokine receptor family, which is located upstream of the JAK-STAT signaling pathway. It is reported that the MPL W515L mutation is present in ~5% of idiopathic myelofibrosis patients and ~1% of essential thrombocythemia patients (7). Previous studies have demonstrated that the MPL W515L mutation is associated with an older age, a lower hemoglobin level and higher platelet counts, however, the association between the mutation and complications, such as thrombosis, is not clear (8,9).

The current report presents the case of an RARS-T patient positive for the MPL W515L mutation, but negative for the JAK2 V617F mutation. To the best of our knowledge, this is the first case study of an MPL W515 mutation in a patient with RARS-T.

## Case report

In August 2012, a 63-year-old female patient was referred to Saint Mary’s Health Center (St. Louis, MO, USA) for the hematological evaluation of macrocytic anemia and thrombocytosis. A complete blood count (CBC) revealed the following results: Hemoglobin, 7.6 g/dl (normal range, 12.0–15.0 g/dl); hematocrit, 24% (normal range, 36.1–44.3%); mean corpuscular volume (MCV), 109 fl (normal range, 80–100 fl); platelet count, 834×10^9^/l (normal range, 150–400×10^9^/l); and a normal white blood cell and differential count 8.2 ×10^9^/l (normal range, 4–10 ×10^9^/l). An iron panel demonstrated an elevated ferritin level (214 mg/l; normal range, 12–150 ng/ml) and increased iron saturation (>95%; normal range, 15–55%). Upon examination, the patient was completely asymptomatic. The patient’s prior CBC, from September 2010, demonstrated similar macrocytic anemia (hemoglobin, 9.5 g/dl; MCV, 102 fl) and thrombocytosis (platelet count, 701×10^9^/l), however, the patient had not been referred for hematological evaluation at that time.

**Figure 1:**
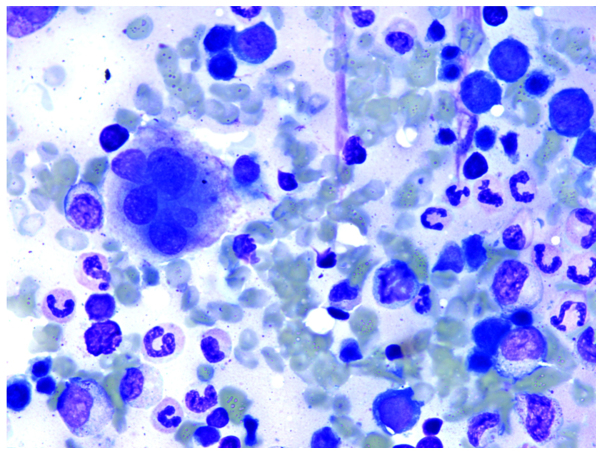
Bone marrow aspirate smear demonstrating hypercellular spicules and trilineage dyspoiesis. Myeloid maturation is mildly dyspoietic and includes hypogranular and hypolobated forms. Erythroid maturation is megaloblastic and megakaryocytes are present in small and monolobated forms (stain, Wright stain; magnification, ×1,000).

**Figure 2:**
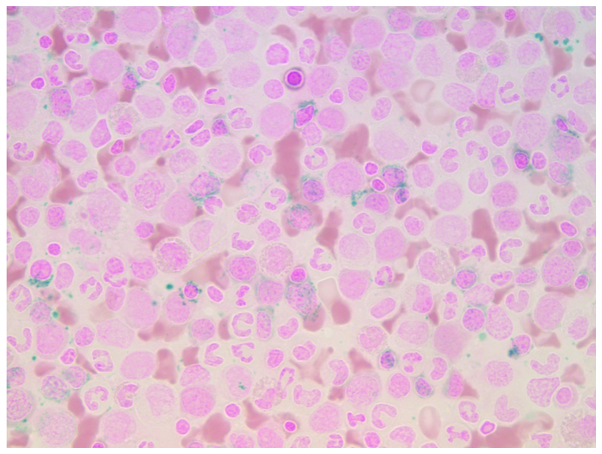
Bone marrow aspirate smear demonstrating an increased number of ring sideroblasts compared with the erythroid precursors (44% of erythroid precursors were ring sideroblasts; staining, Prussian blue; magnification, ×1,000).

A bone marrow aspiration and biopsy demonstrated marked erythroid hyperplasia*,* trilineage dyspoiesis (Fig. 1) and increased ring sideroblasts (Fig. 2) compared with the erythroid precursors; 44% of the precursors were ring sideroblasts. Cytogenetic and fluorescence *in situ* hybridization analysis of the patient was positive for an MPL W515L mutation and an isolated chromosome 13q deletion, however, there was no evidence of a chromosome 5q deletion, JAK2 mutation or BCR-ABL fusion gene. According to the WHO Classification of Tumors (1), the patient was diagnosed with RARS-T, with an MPL W515L mutation, a chromosome 13q deletion and an IPSS Score of 0.5 (intermediate-1 risk).

The patient commenced subcutaneous epoetin α therapy (60,000 units/week) from November 2012, however, following a suboptimal response, epoetin α therapy was terminated and subcutaneous darbepoetin α therapy (300 μg/week) commenced. Furthermore, the patient was administered with 81 mg aspirin per day for the treatment of thrombus prophylaxis. From November 2012 until the writing of this study, the patient’s hemoglobin concentration (range, 8.0–10.0 g/dl) and platelet count (range, 600–770×10^9^/l) have remained stable. At present, the patient is asymptomatic, transfusion-independent, continues to work and maintains a good performance status. A repeat bone marrow biopsy in June 2013 revealed stable hematological results and no evidence of disease progression.

## Discussion

An MPL W515L mutation and isolated chromosome 13q deletion is rare in an RARS-T patient negative for a JAK2 mutation and 5q deletion. A search of the literature reveals a number of studies regarding MPL W515 mutations in MDS with sideroblastic change, however, it does not reveal any studies on MPL W515 mutations in typical RARS-T. Schnittger *et al* (10) reported a case of an MPL W515 mutation with features of ringed sideroblasts and thrombocytosis; however, the patient was not anemic and exhibited an asymptomatic benign course. Another study reported the case of a JAK-2 mutation-negative and MPL W515 mutation-positive patient exhibiting grade 2 myelofibrosis; however, no details regarding clinical course, laboratory results or bone marrow biopys results were provided (3).

The JAK-2 V617F mutation occurs in ~50% of RARS-T patients and appears to be predictive of a lower mortality rate compared with the mutation-negative group (3). A clinical trial of ruxolitinib, an oral JAK-2 inhibitor, is currently ongoing with the aim of investigating its efficacy in the treatment of MDS patients who carry the JAK2 V617F mutation (NCT01895842). MPL, a cellular homologue of the v-MPL oncogene, is located upstream of the JAK-STAT signaling pathway (11). The MPL W515L mutation induces constitutive, cytokine-independent activation of the JAK-STAT signaling pathway, and may be significant in the pathogenesis of RARS-T (12). However, it remains unclear whether the MPL W515L mutation is associated with an improved prognosis in RARS-T patients and whether it acts as a target for JAK2 pathway inhibitors, including ruxolitinib. In the case presented in the current study, the patient remained asymptomatic and transfusion-independent with no disease progression at the one year follow-up, indicating that the MPL W515L mutation is associated with a favorable prognosis. However, further prospective and long-term investigation of patients exhibiting similar cytogenetic profiles is required prior to reaching a definitive conclusion.
